# Substrate complexes of human dipeptidyl peptidase III reveal the mechanism of enzyme inhibition

**DOI:** 10.1038/srep23787

**Published:** 2016-03-30

**Authors:** Prashant Kumar, Viktoria Reithofer, Manuel Reisinger, Silvia Wallner, Tea Pavkov-Keller, Peter Macheroux, Karl Gruber

**Affiliations:** 1 Institute of Molecular Biosciences, University of Graz, Humboldtstraße 50/3, 8010 Graz, Austria; 2 Institute of Biochemistry, Graz University of Technology, Petersgasse 12/2, 8010 Graz, Austria; 3 ACIB – Austrian Centre of Industrial Biotechnology, Petersgasse 14, 8010 Graz, Austria; 4 BioTechMed-Graz, Austria

## Abstract

Human dipeptidyl-peptidase III (hDPP III) is a zinc-dependent hydrolase cleaving dipeptides off the N-termini of various bioactive peptides. Thus, the enzyme is likely involved in a number of physiological processes such as nociception and is also implicated in several forms of cancer. We present high-resolution crystal structures of hDPP III in complex with opioid peptides (Met-and Leu-enkephalin, endomorphin-2) as well as with angiotensin-II and the peptide inhibitor IVYPW. These structures confirm the previously reported large conformational change of the enzyme upon ligand binding and show that the structure of the closed conformation is independent of the nature of the bound peptide. The overall peptide-binding mode is also conserved ensuring the correct positioning of the scissile peptide bond with respect to the catalytic zinc ion. The structure of the angiotensin-II complex shows, how longer peptides are accommodated in the binding cleft of hDPP III. Differences in the binding modes allow a distinction between real substrates and inhibitory peptides or “slow” substrates. The latter displace a zinc bound water molecule necessitating the energetically much less favoured anhydride mechanism as opposed to the favoured promoted-water mechanism. The structural data also form the necessary framework for the design of specific hDPP III inhibitors.

Human dipeptidyl peptidase III (hDPP III), also known as enkephalinase B, is a member of the M49 family of zinc dependent metallopeptidases and cleaves dipeptides sequentially from the N-terminus of various bioactive peptide substrates^1^,^2^. *In vitro*, DPP III shows a high affinity and ability to cleave opioid peptides such as enkephalins and endomorphins^3^,^4^. These opioid peptides together with their receptors constitute the endogenous opioid system and regulate a plethora of diverse physiological functions in mammals, such as signal transduction, endocrine and immune function, gastrointestinal motility, complex social behaviour (*e.g.* sexual activity), vulnerability to drug addiction and most importantly pain modulation (nociception)^5^,^6^. Thus, the enzyme could itself potentially be involved in affecting a large number of physiological processes.

Other, recent data also indicate the involvement of DPP III in cancer. It has been shown that DPP III competes with NRF2 to interact with KEAP1 ubiquitin ligase, thus blocking the ubiquitination of NRF2, leading to NFR2 mediated uncontrolled transcriptional activation especially in squamous cell lung carcinoma^7^. The enzyme has also been found to be overexpressed in ovarian malignant tissues, endometrial carcinoma^8^ and glioblastoma cells^9^. Some reports have also suggested its involvement in cataractogenesis^10^ and in the endogenous defence against oxidative stress by activating the anti-oxidant response element through nuclear translocation of NRF2^11^.

In mammals, DPP III is widely distributed in several tissues such as erythrocytes, brain, spinal cord, skin, eye lens extracts, kidney, liver and small intestine^1^. It was also found to be a component of the human central proteome, a set of 1124 proteins ubiquitously and abundantly expressed in all human cell lines^12^. In addition, DPP III orthologs have also been identified in lower eukaryotes (yeast) as well as some bacterial species^13^. DPP III is mostly characterized as a cytosolic protein, but its membrane association has also been reported in rat brain^14^, in cortical lipid rafts of a murine model of Alzheimer’s disease^15^ and in *Drosophila melanogaster*^16^.

Crystal structures have so far been determined for DPP III from humans^17^ as well as from *Saccharomyces cerevisiae*^18^. Despite the low sequence identity (36%) shared by the two proteins, both structures are very similar and consist of two lobes separated by a wide cleft (Fig. 1a). The “upper” lobe is mostly α-helical and contains the zinc binding site as well as the catalytically important residues. In both structures, two histidine residues (His-450 and His-455 in the human enzyme) and a glutamate (Glu-508) coordinate the zinc ion. A water molecule completes the tetrahedral coordination of the metal ion. The zinc coordinating residues are part of conserved sequence motifs (450-HELLGH-455 and 507-EECRAE-512) characteristic for members of the family M49 metallopeptidases. Another glutamate (Glu-451) from the HELLGH-motif has been proposed to act as a general base activating the water molecule, which attacks the scissile peptide bond^19^. This is similar to the catalytic mechanism proposed for other zinc dependent proteases such as thermolysin^20^,^21^. Indeed, the exchange of Glu-451 by alanine in rat DPP III rendered the enzyme inactive^19^. The lower lobe exhibits a mixed α- and β-fold with a five-stranded β-barrel forming the structural core of this lobe (Fig. 1a). The two lobes are connected by a number of quite flexible loop regions.

A large conformational change leading to the closure of the cleft was observed in the structure of human DPP III in complex with the synthetic opioid peptide tynorphin (VVYPW, Fig. 1b)^17^. Peptide binding to DPP III was shown to be driven by entropic changes and the release of ordered water molecules from the binding cleft is proposed to be mainly responsible for the entropy increase^17^. The peptide itself primarily interacts via its main chain with the lower lobe (especially with the five-stranded β-barrel) and the domain motion is necessary to bring the scissile peptide group in proximity to the catalytic machinery of the enzyme.

DPP III exhibits a broad substrate specificity and has been shown to bind and cleave a number of different lengths peptides (ranging from tetra- to decapeptides) almost irrespective of their amino acid composition^1^. While the tynorphin complex provided a first glimpse on the peptide binding mode, it is still unclear, whether this binding mode is conserved for different peptides, whether the same domain motion is induced by the binding of different ligands and how longer peptides (octa- to decapeptides) are accommodated in the binding cleft. Tynorphin itself has originally been described as an inhibitor of human DPP III, but it appears to be hydrolysed over a period of about 24 hours^22^. In contrast to that, the enzyme cleaves other peptides very efficiently leading to the question, whether the binding mode is different between true substrates and inhibitory peptides.

In this study, we therefore determined crystal structures of hDPP III in complex with a number of different peptides – Leu-enkephalin (YGGFL), Met-enkephalin (YGGFM), endomorphin-2 (YPFF-NH2), angiotensin-II (DRVYIHPF) as well as the synthetic opioid peptide IVYPW. We also determined the structure of the unbound enzyme in the closed conformation. These structures show that the general peptide binding mode as well as the large domain motion is conserved. They also indicate strategies for binding longer peptides (such as angiotensin-II) and hint at differences in the binding of substrates *vs.* inhibitors (or “slow” substrates), which can be interpreted regarding the catalytic mechanism of DPP III.

## Results and Discussion

We determined crystal structures of the inactive E451A variant of human dipeptidyl peptidase III in complex with different peptides with resolutions ranging from 1.84 to 2.76 Å (Table 1). The co-crystallized ligands comprised known *in vitro* substrates of hDPP III, such as Met- and Leu-enkephalin, endomorphin-2 and angiotensin-II, as well as the synthetic opioid peptide IVYPW, which has been described as an inhibitor of the enzyme^23^.

The specific hDPP III construct used for crystallization trials lacks 11 C-terminal amino acid residues, which had been predicted to be disordered. In addition, a number of cysteine residues on the surface of the enzyme were replaced by serines, while two other surface residues were replaced by cysteines (see Methods). This construct was originally designed for single molecule fluorescence energy transfer experiments (smFRET) in solution, but we also used it for crystallization, because we were unable to reproduce crystallization with the hDPP III construct used in previous studies^17^. Interestingly, one of the newly introduced cysteine residues (Cys-207) apparently forms an intermolecular disulfide bridge in the crystal (Supplementary Figure S2), which may promote crystallization. We also tested this construct regarding its enzymatic activity using Arg-Arg-β-naphthylamide as substrate and observed no significant differences to the wild-type enzyme. In our assays, we obtained a k_cat_ of 24 s^−1^ and a K_M_ of 24 μM for the wild-type enzyme, whereas we observed a k_cat_ of 28 s^−1^ and a K_M_ of 30 μM for the active smFRET construct (still containing residue Glu-451).

### Overall structures

All crystals presented here were isomorphous (monoclinic, *C*2) and contained one hDPP III molecule per asymmetric unit. An analysis of intermolecular interactions within the crystals using the PISA server^24^ indicated that the enzyme is most likely present as a monomer in solution. In all complex structures, hDPP III exhibits a closed conformation equivalent to what has previously been observed in the complex with tynorphin (VVYPW)^17^. The different protein chains superimpose well onto each other with an average root-mean-square-deviation (rmsd) for Cα-atoms of 0.25 Å (Supplementary Figure S3).

**Figure 1:**
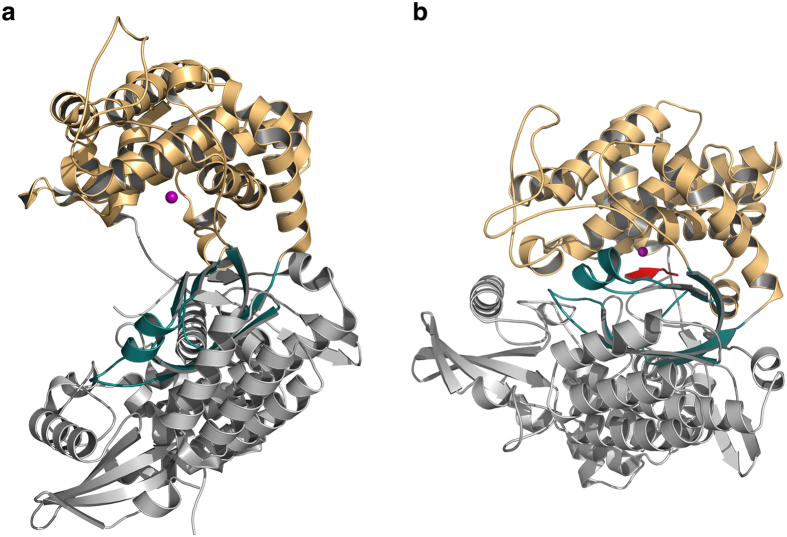
Overall structure of human dipeptidyl peptidase III. (**a**) Cartoon representation of the structure of the unbound hDPP III. The upper lobe is shown in orange, the lower lobe in grey and the five-stranded β-core in deep teal. The catalytic zinc ion is depicted as a magenta sphere. (**b**) Cartoon representation of the closed conformation of hDPP III. The bound peptide is shown in red. The figure was prepared using the program PyMol (http://www.pymol.org/).

All studied peptide ligands also bind in the same location in the inter-lobe cleft of hDPP III (Supplementary Figure S4). In all cases, the N-terminal residues of the peptides form mostly extended conformations and the main chains interact with the five-stranded β-barrel of the lower lobe (Fig. 1b) in an antiparallel fashion. This binding mode is quite common for peptides and optimizes the backbone interactions through hydrogen bonds^25^. The dipeptidase specificity (exclusive cleavage of the peptide after the second residue) can be rationalized by the correct positioning of the N-terminus of the peptide and the catalytic zinc ion. Despite the similarities, there are subtle differences between some of the complex structures (*e.g.* for the proline containing endomorphin-2), which will be discussed in more detail below.

In the previously determined structure of the complex with tynorphin^17^ electron density was missing for the catalytic zinc ion. The same is true for the present structure of the complex with angiotensin II. In both cases, crystallization took several months and the zinc ion probably got sequestered over the long time by the phosphate present in the crystallization buffer. In the other complex structures, the electron density for zinc was clearly defined indicating a high occupancy of this position. In these cases the crystals were grown over a much shorter period of a couple of weeks after seeding.

As a serendipitous result of the co-crystallization trials we also determined the structure of the unbound enzyme in the closed conformation. As yet, structural studies on human and yeast DPP III in the absence of ligands^17^,^18^ have always yielded an open extended conformation (Fig. 1a). Because the crystals in this study were obtained through seeding with microcrystals of an hDPP III ligand complex (see Methods), it is very likely that this procedure induced crystallization of the protein in the closed conformation.

### Complexes with Met- and Leu-enkephalin

**Figure 2:**
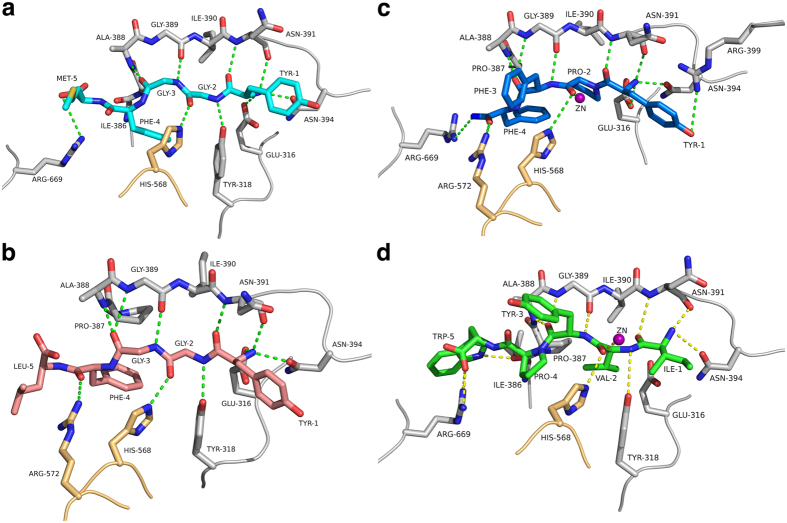
Structures of opioid peptide complexes. (**a**) Interactions of Met-enkephalin with hDPP III. The bound peptide is shown in cyan. Amino acid residues from the upper and lower lobe of hDPP III are shown in orange and grey, respectively. Dashed lines represent potential hydrogen bonding interactions. (**b**) Interactions with Leu-enkephalin (pink). (**c**) Interactions with endomorphin-2 (blue). (**d**) Interactions with the synthetic opioid peptide IVYPW (green). A magenta sphere represents the zinc ion in panels c and d. The figure was prepared using the program PyMol (http://www.pymol.org/).

Met-enkephalin (YGGFM) and Leu-enkephalin (YGGFL) are endogenous opioid neurotransmitters which are found in the brains and spinal cords of many animals, including humans, and which have been shown to be cleaved by hDPP III^1^. In addition, Leu-enkephalin has been shown to bind to the inactive hDPP3 variant with a *K*_d_ of 3.6 μM^17^. In the complex structures, the N-termini of both peptides are anchored to the protein through hydrogen bonding and electrostatic interactions from the side chains of Tyr-318, Glu-316 and Asn-394 (Fig. 2a,b, Supplementary Figures S5 and S6). As mentioned above, the first three amino acids form a β-strand, which interacts with the five-stranded β-core in an antiparallel fashion (through hydrogen bonds to Ala-388 to Asn-391). These interactions stabilize the extended conformation of the peptide, which would otherwise not be favoured due to the glycine residues at positions 2 and 3 of the enkephalins. As in the case of the tynorphin complex, most interactions with the backbone of the peptide ligand are formed with residues from the lower lobe. One exception is His-568 from the upper lobe, which is hydrogen bonded to the carbonyl oxygen of the scissile peptide bond. This histidine residue is conserved among DPP IIIs and the observed interaction has also been implied to be important in the mechanism of DPP III^18^ as well as in other metalloproteases such as thermolysin^20^,^21^ for the stabilization of the oxyanion intermediate.

In the complex with Leu-enkephalin an additional residue (Arg-572) from the upper lobe forms a hydrogen bond with of Phe-4 of the peptide (3.3 Å, Fig. 2b). In the case of Met-enkephalin, this distance is slightly larger (4.2 Å). In both complexes the C-terminal residue (Met or Leu) appears to be quite flexible as indicated by weaker electron density in this region (Supplementary Figure S1). Therefore, the differences in the interactions of the C-terminal residues of the enkephalins in the complex structures (Fig. 2a,b) may not be significant.

Quite unexpectedly, the zinc ion does not directly interact with the P1 carbonyl of the peptide (the scissile peptide) but is at a distance of 3.7 Å and 3.6 Å for Met and Leu-enkephalin, respectively. Instead, a water molecule completes the tetrahedral coordination of the metal ion.

### Complex with endomorphin-2

Endomorphins are more recently identified opioid peptides that act as a neurotransmitter/neuromodulators in mammals^26^,^27^. Their effects involve antinociceptive action and vasodilation of the endothelium^28^. There are two types of endomorphins, which differ in one amino acid: endomorphin-1 (YPWF-NH2) and endomorphin-2 (YPFF-NH2). Both endomorphins have amidated C-termini. Proline containing peptides are more resistant to cleavage by peptidases, however hDPP III has been shown to act as a post-proline peptidase^3^ and to bind endomorphins with micromolar affinity^17^.

Endomorphin-2 binds to hDPP III similar to other peptides (Fig. 2c and Supplementary Figure S7). Again, the N-terminus is bound by polar interactions with the conserved residues Asp-316, Asn-391 and Asn-394. Due to the proline at position 2, however, endomorphin-2 cannot form the canonical antiparallel β-strand interaction and the otherwise conserved interaction with Tyr-318 is missing. Also, His-568 is too far away (3.7 Å) in the present structure to form a hydrogen bond with the carbonyl group of the Pro-2. In contrast to the complexes with Met- and Leu-enkephalin, the carbonyl group of Pro-2 is coordinating the catalytic zinc ion.

### Complex with IVYPW

Similar to tynorphin (VVYPW), which is a derivative of spinorphin^29^ and has been shown to inhibit partially purified DPP III from monkey brain^22^, IVYPW inhibited rat DPP III with nanomolar affinity^23^. IVYPW was in fact found to be a stronger inhibitor than tynorphin.

In the present structure, IVYPW binds in the same mode as VVYPW^17^ and the other peptides discussed so far including the formation of the antiparallel β-strand interaction and the hydrogen bond from His-568 (Fig. 2d and Supplementary Figure S8). In addition, the zinc ion is coordinated by the carbonyl oxygen of Val-2 similar as in the complex with endomorphin-2.

### Complex with angiotensin-II

**Figure 3:**
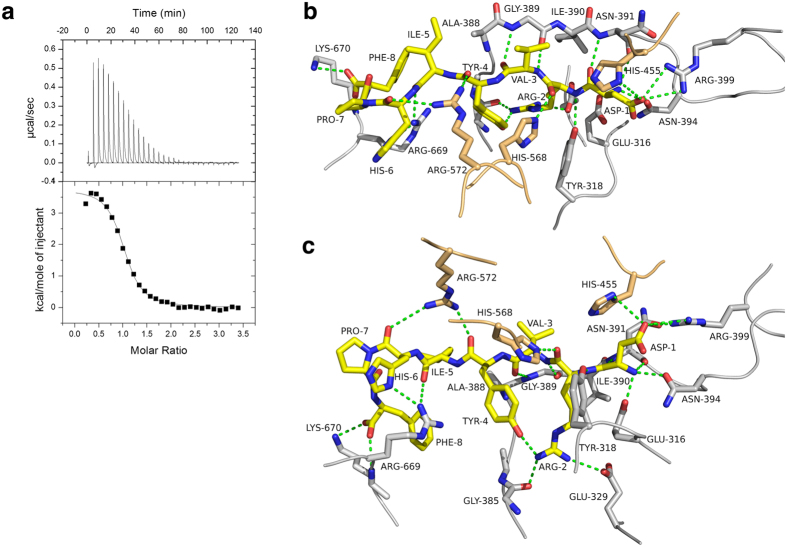
Binding of angiotensin-II to hDPP III. (**a**) Isothermal titration calorimetry thermogram of the titration of angiotensin-II into a solution of the inactive hDPP III E451A-variant. The solid curve represents the best fit using a “one-binding-site” model yielding the following thermodynamic parameters: K_d_ *=* 1.64 ± 0.12 μM, Δ*H* *=* 15.25 ± 0.17 kJ/mol, Δ*G* *=* −32.93 ± 0.12 kJ/mol, *T*Δ*S* *=* 48.18 ± 0.24 kJ/mol. (**b**,**c**) Two different views (related by an about 90° rotation around the horizontal axis) of the Interactions of angiotensin-II with hDPP III. The bound peptide is shown in yellow. Amino acid residues from the upper and lower lobe of the enzyme are shown in orange and grey, respectively. Dashed lines represent potential hydrogen bonding interactions. Panels (**b**,**c**) were prepared using the program PyMol (http://www.pymol.org/).

The octapeptide angiotensin-II (DRVYIHPF) is a potent vasoconstrictor and is involved in regulating mammalian blood pressure. Although angiotensin-II is not an opioid peptide, DPP III has been shown to cleave angiotensin-II *in vitro*^4^,^14^. We performed isothermal titration calorimetry (ITC) to determine the binding affinity of angiotensin-II to the inactive hDPP III variant. Similar to tynorphin and other opioid peptides binding to hDPP III is an endothermic process driven by entropic changes (Fig. 3a). The dissociation constant (*K*_*d*_) calculated from the ITC data was 1.64 μM.

The main reason for us to co-crystallize hDPP III with angiotensin-II was to get some idea, how longer peptides bind to the enzyme. As in the previous examples, the N-terminus of the peptide forms extensive polar contacts with the conserved residues Asn-394, Glu-316 and Tyr-318 and is involved in β-type interactions with the core of the protein (Fig. 3b,c, Supplementary Figure S9). The side chain of Asp-1 of the peptide forms a salt bridge with Arg-399 and is additionally hydrogen bonded to His-455 from the conserved HELLGH zinc-binding motif. His-568 interacts with the carbonyl of Arg-2 of angiotensin-II, again supporting the hypothesis of oxyanion stabilization during catalysis. The guanidinium group of Arg-572 forms hydrogen bonds with carbonyl groups of Tyr-4 and His-6 of angiotensin-II.

In order to be accommodated in the binding cleft, angiotensin-II adopts a turn-like conformation with a cis-peptide formed between His-6 and Pro-7 (Fig. 3b,c and Supplementary Figure S9). This is consistent with previously determined structures of angiotensin-II in aqueous solution^30^. The C-terminus of the peptide is stabilized by polar interactions with Arg-669 and Lys-670. Arg-669 forms a hydrogen bond with the carbonyl group of Ile-5, whereas Lys-670 forms a salt bridge with the C-terminal carboxylate of the ligand. It appears that the binding cleft is voluminous enough to accommodate a heptapeptide in a more or less extended conformation, whereas longer peptides have to bend as seen in the angiotensin-II complex (Supplementary Figure S10).

### Mechanistic implications

Peptides like enkephalins have been shown to be hydrolysed efficiently by hDPP III (at least *in vitro*), whereas other, similar length peptides, such as tynorphin or IVYPW, inhibit the enzyme or at least get hydrolysed only very slowly. The overall binding mode of peptides as seen in the available complex structures does so far not allow a clear distinction between substrates and inhibitors (“slow” substrates).

**Figure 4:**
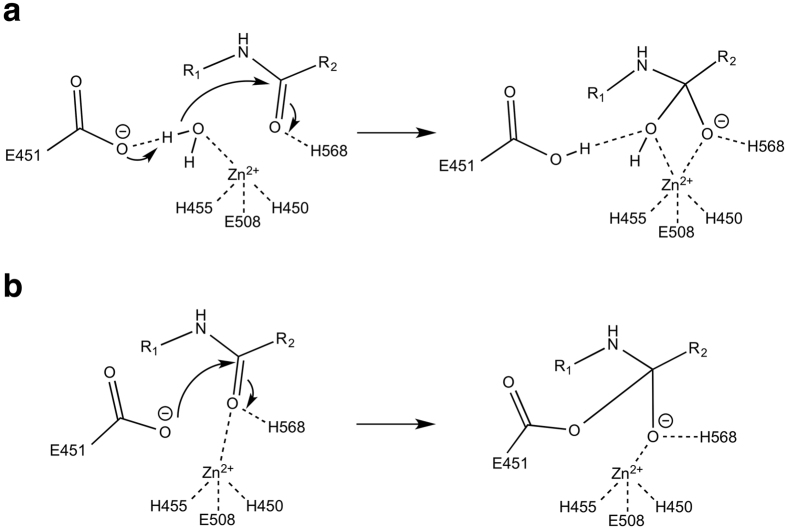
Possible mechanisms of peptide hydrolysis catalysed by hDPP III. (**a**) Promoted-water mechanism. (**b**) Anhydride mechanism.

**Figure 5:**
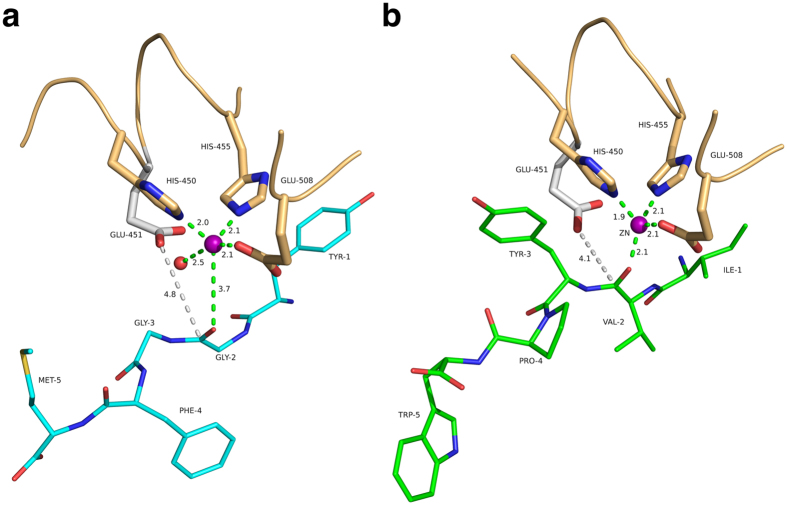
Coordination of the catalytic zinc ion in substrate and inhibitor complexes. (**a**) Complex with Met-enkephalin (cyan). The zinc ion is represented as a magenta sphere; the Zn-bound water molecule is shown as a red sphere. Interactions with the metal ion are shown as dashed lines. The side chain of Glu-451 shown in grey (not present in the inactive E451A-variant) was modelled based on structure of the unbound enzyme 17. Potential interactions with the zinc ion are shown as green dashed lines. The distance between the carboxylate of the modelled Glu-451 and the carbonyl carbon of the scissile peptide bond is indicated as grey dashed lines. (**b**) Complex with the synthetic opioid peptide IVYPW (green). The figure was prepared using the program PyMol (http://www.pymol.org/).

Based on the similarity of the zinc binding motif and the active site of yeast DPP III with other metallopeptidases, Baral *et al.* proposed a catalytic mechanism^18^, which very likely also applies to the human enzyme. This mechanism involves activation of the scissile amide and the attacking water molecule by the zinc ion. In addition, the water is activated by deprotonation trough a general base (Glu-451) and the resulting tetrahedral intermediate (oxyanion) is stabilized by the interaction with His-568 (Fig. 4a). When analysing the interactions with the catalytic zinc ion, we observed that the carbonyl group of the scissile peptide bond is not directly interacting with the catalytic zinc ion (3.7 Å distance) in the complexes with Met- and Leu-enkephalin and a water molecule is bound to the ion (Fig. 5a). Instead, the metal ion is coordinated by the respective carbonyl group in the complex with the inhibitory IVYPW and there is no room for an additional water molecule.

Figure 5 also shows the side chain of Glu-451 modelled based on a superposition of the complex structures with the structure of unbound, active hDPP III. In the complex with Met-enkephalin the zinc bound water molecule is situated right between the side chain of Glu-451 and the scissile amide bond (Fig. 5a). Despite the uncertainties regarding the exact conformation of Glu-451, this configuration can be considered structurally similar to the Michaelis complex and the water molecule is in a suitable position for the nucleophilic attack on the peptide group.

In the inhibitor (IVYPW) complex, on the other hand, the water molecule is missing. The modelled side chain of Glu-451 is about 4 Å from the scissile peptide bond (Fig. 5b) and thus could directly act as the nucleophile leading to the formation of an acyl-enzyme-like intermediate (Fig. 4b). Such a mechanism would be similar to the proposed anhydride pathway of peptide hydrolysis by the zinc dependent enzyme carboxypeptidase A^31^,^32^. Even though carboxypeptidase A (CPA) is an exopeptidase, it shares similar active site arrangements to hDPP III and thermolysin. The anhydride pathway has been contested as the preferred pathway for peptide hydrolysis, because it includes a high energy acyl-enzyme intermediate as assessed by quantum chemical calculations^33^. There is also experimental evidence favouring the water-mediated pathway. Results from ^18^O labelling experiments suggest that the acyl-enzyme intermediate is unlikely to form in the hydrolysis of peptides by CPA^34^,^35^. There is, however, evidence supporting the formation of acyl-enzyme intermediate in ester hydrolysis by CPA at low temperatures^36^,^37^,^38^. It has also been proposed that ester and peptide hydrolysis might not necessarily be the same and that they may follow different paths, because esters bind directly to the metal ion, whereas peptides do not^39^. Several CPA-inhibitor complexes indicate that a direct metal carbonyl interaction represents a non-productive binding mode preventing the activation of the water nucleophile^39^. Based on the similarity between CPA and hDPP III, we hypothesize that inhibitory peptides, such as IVYPW and tynorphin, might displace the water molecule originally bound to the zinc ion. In this case the reaction may still follow the energetically unfavourable and therefore slow anhydride pathway (Fig. 4b). In the case of efficiently cleaved peptides like the enkephalins, on the other hand, the reaction follows the energetically favoured and therefore faster water mediated hydrolysis pathway (Fig. 4a). The exact reason for the preference of a particular peptide sequence for one of the two binding modes (“zinc-on” or “zinc-off”) is still obscure and further studies are necessary, *e.g.* regarding the molecular dynamics of DPP III peptide complexes.

## Conclusion

We determined and analysed several crystal structures of complexes of human dipeptidyl peptidase III with different peptides. The structures confirm the previously reported large conformational change of the enzyme upon ligand binding. Although hDPP III binds to a wide variety of peptides with different sequences and lengths (tetra- to decapeptides), the structure of the closed conformation appears to be independent of the nature of the bound peptide. Similarly, the overall binding mode of peptides to hDPP III is also conserved, although subtle changes in the specific interactions are observed. Common to all complex structures are extensive polar contacts of the N-terminal peptide residues and the formation of β-type interactions with the core of the enzyme. These interactions ensure the correct positioning of the scissile peptide bond with respect to the catalytic zinc ion. The structure of the angiotensin-II complex shows for the first time, how longer peptides are accommodated in the binding cleft of hDPP III. Because the enzyme has been shown to act upon bioactive and opioid peptides, the structural data also form the necessary framework for the design and discovery of specific DPP III inhibitors.

The present crystal structures also allow hypotheses regarding the catalytic mechanism of hDPP III and regarding the differentiation of “good” substrates from inhibitors or “slow” substrate. Our data suggest that the two groups differ in the interaction of the carbonyl group of the scissile peptide bond with the catalytic zinc ion. Inhibitory peptides appear to displace a zinc bound water molecule necessitating an energetically much less favoured anhydride mechanism compared to the favoured water mediated hydrolysis mechanism.

## Methods

### Protein expression and purification

The gene encoding a C-terminally truncated version of the inactive E451A variant of hDPP III_(1–726)_ used in this study was cloned into the expression vector pET28-MHL, which includes an N-terminal fusion sequence encoding a His_6_-tag and a Tobacco Etch Virus (TEV) protease cleavage site, as described previously^17^. The truncation of 11 C-terminal amino acid residues was performed to improve the crystallizability of the protein as those amino acids were predicted to be unordered and unfavourable for crystallization. The specific construct used for the crystallization trials has originally been produced for single molecule fluorescence resonance energy transfer experiments (smFRET). Therefore, three surface accessible cysteine residues (C19, C519 and C654) were exchanged to serine and two solvent accessible residues E207 and S491 were exchanged to cysteines (sites for the attachment of the fluorescence dyes). All these amino acid exchanges were performed by site directed mutagenesis with the inactive variant E451A pET-28MHL-hDPP3_1–726_ as template. The plasmid containing the desired mutations, confirmed by sequencing results, was then transformed into a BL21-CodonPlus (DE3) RIL strain. The cell culture was grown in Luria-Bertani (LB) medium containing 50 μg mL^−1^ kanamycin. Enzyme expression was induced with 0.4 mM isopropyl-1-thio-D-galactopyranoside (IPTG) after the culture medium reached an OD of 0.6–0.8. After being allowed to grow overnight at 18 °C, the cells were harvested by centrifugation at 4000 *g* for 10 min. The harvested cell pellet was resuspended in 50 mM Tris-HCl pH 8.0, 300 mM NaCl, 10 mM imidazole and 0.05% 2-mercaptoethanol (lysis buffer) and lysed by sonication. Centrifugation at 25000 *g* for 45 min at 4 °C was performed to remove cell debris and the supernatant was subjected to affinity chromatography on Ni-NTA resin (5 mL prepacked His-trap FF, GE Healthcare) previously equilibrated with lysis buffer. After washing, bound protein was eluted using lysis buffer containing 500 mM imidazole. Anion exchange chromatography was performed to improve the purity of the sample using a prepacked ResourceQ column (GE Healthcare). The column was equilibrated with 20 mM Tris-HCl pH-8.0, 0.05% of 2-mercaptoethanol and the bound protein was eluted using a gradient of the same buffer containing 1 M NaCl. The pure fractions were pooled and incubated with TEV protease overnight at 4 °C to cleave off the His_6_-tag. After TEV cleavage the protein was again passed through a Ni-NTA column and the flow through containing cleaved protein was collected and pooled. This sample was then applied to a Superdex 200 26/60 gel filtration column (GE Healthcare) and pure fractions corresponding to a molecular weight of ~82 kDa were collected and concentrated. The buffer used for gel filtration was 100 mM multi component buffer (L-malic acid, MES and Tris, pH-8.0)^40^, 100 mM NaCl and 1 mM tris(2-carboxyethyl)phosphine (TCEP). The purity of the fractions was analysed by 12% SDS-PAGE.

### Activity assay

The enzymatic activities of the wild-type enzyme and the active smFRET construct (still containing residue Glu-451) were determined using a fluorometric assay, which measures the release of β-naphthylamine upon cleavage of the synthetic substrate Arg-Arg-β-naphthylamide (excitation at 320 nm and emission at 420 nm)^41^. Kinetic parameters were determined at 37 °C in 50mM Tris-HCl buffer pH 8.0, containing 100 mM NaCl. The protein concentrations were 20 pM (wild-type enzyme) and 50 pM (smFRET construct). The substrate concentration was varied from 1 μM to 200 μM. The initial rate of fluorescence increase was monitored continuously for 5 min and was converted into the velocity of the reaction using a calibration curve for β-naphthylamine. Non-linear regression using the program GraphPad Prism (www.graphpad.com) yielded the values for v_max_ and K_M_.

### Crystal structure determination

For all co-crystallization trials, the protein samples were concentrated to 8 mg mL^−1^. In each case the protein:ligand ratio was approximately 1:30. For this purpose all peptide ligands were dissolved in the same multicomponent buffer as used in the final purification step. In the beginning, we were unable to reproduce the published crystallization conditions (0.056 M sodium phosphate monobasic monohydrate, 1.344 M potassium phosphate dibasic, pH 8.2). However, we obtained diffraction quality monoclinic crystals of the protein construct prepared for the smFRET measurements in complex with angiotensin II after 2 months. From these crystals, a seeding stock was prepared and microseeding was performed in the other co-crystallization setups with different peptide ligands (Met-enkephalin, Leu-enkephalin, endomorphin-2 and IVYPW). Crystallization trials were conducted at 20 °C using sitting drop vapour diffusion. The total drop volume was 1.2 μL with a protein:reservoir:seeds ratio of 60:40:20. Microseeding led to a significant speed up of the crystallization process as diffraction quality co-crystals with all peptide ligands appeared within a week. These crystals were allowed to grow for a month before being flash-cooled in liquid nitrogen without any additional cryoprotectant.

The data were processed using the program XDS^42^ and the structures were solved by molecular replacement using the program PHASER^43^ with the structure of tynorphin bound hDPP III_(1–726)_ as search model. The structures were further refined using the program PHENIX^44^. The program COOT^45^ was used for model fitting and real space refinement using σ_A_-weighted 2F_o_-F_c_ and F_o_-F_c_ electron density maps. R_free_-values^46^ were computed from 5% randomly chosen reflections not used for refinement. For all structures except the complex with angiotensin-II clear electron density for the catalytic zinc ion was observed. Additional larger electron density peaks were interpreted as potassium and magnesium ions in all structures. Potassium was present in the crystallization buffer, whereas the magnesium ions might already be bound to the protein in the cell, because neither the purification buffers nor the crystallization condition contained any magnesium. The electron densities for the bound ligands were also reasonably well defined in most cases (Supplementary Figure S1), although the C-terminal residues of Met- and Leu-enkephalin were found to be flexible and the occupancy of endomorphin-2 had to be reduced to 0.8. The stereochemistry of the structures was checked using Molprobity^47^ showing one Ramachandran plot outlier (Ser-500) in each structure. Details of the data collection, processing and structure refinement are summarized in Table 1.

### Isothermal titration calorimetry

Microcalorimetric data for the binding of angiotensin II to hDPP3_1–726_ E451A (in 50 mM Tris-HCl pH 8.0, 100 mM NaCl) were measured at 25 °C with a VP-ITC microcalorimeter (MicroCal, Northampton, MA, USA). For this purpose, the eight amino acid peptide angiotensin II (DRVYIHPF) was also dissolved in the same buffer and both solutions were degassed immediately before the measurement. A titration experiment consisted of 30 injections of an 800 μM solution of angiotensin II in the syringe against a 40 μM solution of the enzyme in the sample cell. In a typical experiment, a total of one aliquot of 2 *μ*L and 29 aliquots of 10 *μ*L of the peptide solution were injected with a rate of 0.5 μL/s into 1.421 mL of the protein solution under constant stirring at 270 rpm. Every injection was carried out over a period of 20 s with a spacing of 225 s between the injections. The corresponding heat of binding was calculated by integrating the area under each observed peak in the thermogram. A reference measurement (peptide injected into buffer) was performed in the same way and was subtracted from the experiment to correct for the heat of dilution of the peptide. The corrected values were then plotted against the ratio of peptide *vs.* protein concentration in the cell to generate the binding isotherm. Nonlinear least squares fitting using Origin version 7.0 (MicroCal) was used to obtain association constants (*K*_*a*_), binding enthalpies (Δ*H*) as well as the binding stoichiometry. *K*_*a*_ and Gibbs free energy (Δ*G*) are related by *ΔG* *=* *−RT*ln*(K*_*a*_ ) *=* Δ*H* - *T*Δ*S*, where *ΔG, ΔH* and *ΔS* are the changes in free energy, enthalpy and entropy of binding, respectively. *T* is the absolute temperature and *R* is the ideal gas constant (8.314 J mol^−1^ K^−1^).

## Additional Information

**Accession codes:** Coordinates and structure factors have been deposited with the PDB under accession codes 5E33, 5E3A, 5E2Q, 5EHH, 5E3C and 5EGY.

**How to cite this article**: Kumar, P. *et al.* Substrate complexes of human dipeptidyl peptidase III reveal the mechanism of enzyme inhibition. *Sci. Rep.* **6**, 23787; doi: 10.1038/srep23787 (2016).

## Supplementary Material

Supplementary Information

## Figures and Tables

**Table 1:** Data collection and refinement statistics.

	Met-enkephalin	Leu-enkephalin	angiotensin-II	endomorphin-2	IVYPW	unbound
Data collection						
Beamline	ESRF-ID29	ESRF-ID29	ESRF-BM14U	ESRF-ID29	ESRF-ID29	ESRF-BM14U
Wavelength (Å)	0.972	0.972	0.954	0.972	0.972	0.979
Space group	*C*2	*C*2	*C*2	*C*2	*C*2	*C*2
Unit cell (Å, °)	119.79, 105.76, 65.17, β=93.5	119.55, 105.75, 64.99, β=93.4	119.13, 105.92, 64.84, β=93.9	120.04, 105.46, 64.72, β=93.5	120.65, 106.39, 65.12, β=93.4	119.19, 106.20, 62.57, β=93.8
Resolution (Å)	45.42–1.84 (1.90–1.84)*	39.58–2.05 (2.12–2.05)*	45.33–2.40 (2.49–2.40)*	49.09–2.38 (2.46–2.38)*	45.56–2.76 (2.86–2.76)*	43.83–2.74 (2.83–2.74)*
Total reflections	231251 (22436)	144509 (14714)	109877 (6613)	110082 (10317)	78751 (6542)	67415 (6257)
Unique reflections	68276 (6626)	48267 (4804)	30464 (2628)	32020 (3068)	20651 (1778)	20281 (1977)
R_merge_	0.051 (0.737)	0.088 (0.619)	0.105 (0.577)	0.091 (0.632)	0.211 (0.501)	0.072 (0.794)
<*I*/σ(*I*)>	14.78 (1.75)	10.73 (1.85)	9.88 (1.73)	10.61 (1.78)	4.39 (1.44)	12.01 (1.64)
R_pim_	0.033 (0.55)	0.064 (0.40)	0.065 (0.429)	0.058 (0.377)	0.122 (0.272)	0.048 (0.48)
Multiplicity	3.4 (3.4)	3.0 (3.1)	3.6 (2.5)	3.4 (3.4)	3.8 (3.7)	3.3 (3.2)
Completeness (%)	96.9 (93.8)	95.1 (95.5)	97.4 (84.2)	98.9 (95.4)	97.9 (84.9)	98.8 (96.0)
Wilson B-factor (Å^2^)	30.1	33.0	34.7	40.6	57.4	69.0
CC_1/2_	0.999 (0.628)	0.995 (0.705)	0.994 (0.659)	0.996 (0.660)	0.926 (0.734)	0.998 (0.707)
CC^*^	1.000 (0.878)	0.999 (0.909)	0.998 (0.891)	0.999 (0.892)	0.981 (0.920)	0.999 (0.910)
Refinement						
R_work_	0.1815	0.2088	0.2062	0.1938	0.2040	0.2058
*R*_free_	0.2237	0.2599	0.2473	0.2370	0.2528	0.2462
Nr. of non-H atoms	6339	6165	6000	6059	5835	5761
Nr. of water molecules	519	363	165	270	29	22
Avg. B-factor (Å^2^)	37.0	37.2	38.5	37.4	56.9	85.6
protein/peptide	37.1	37.4	38.6	37.6	56.3	85.6
metal ions	28.5	30.2	30.8	30.0	53.2	78.5
solvent	37.0	34.4	33.8	32.5	45.4	76.7
Rmsd bond lengths (Å)	0.011	0.002	0.002	0.005	0.004	0.002
Rmsd bond angles (°)	1.23	0.62	0.58	0.71	0.58	0.55
Clash score	5.82	3.05	2.77	4.37	3.83	3.96
Ramachandran outl.	1	1	0	0	1	2
PDB-entry	5E33	5E3A	5E2Q	5EHH	5E3C	5EGY

^*^values in parentheses represent the highest resolution shell.
