# Supplementary material for: Substrate complexes of human dipeptidyl peptidase III reveal the mechanism of enzyme inhibition
Source: Sci Rep. 2016 Mar 30;6:23787. doi: 10.1038/srep23787 (PMC4824452; doi:10.1038/srep23787)
Supplement: Supplementary Information [file srep23787-s1.pdf]

# Substrate complexes of human dipeptidyl peptidase III reveal the mechanism of enzyme inhibition

Prashant Kumar[1], Viktoria Reithofer[1], Manuel Reisinger[1], Silvia Wallner[2], Tea Pavkov-Keller[3], Peter Macheroux[2,4] and Karl Gruber[1,4*]

[1]Institute of Molecular Biosciences, University of Graz, Humboldtstraße 50/3, 8010 Graz, Austria

[2]Institute of Biochemistry, Graz University of Technology, Petersgasse 12/2, 8010 Graz, Austria

[3]ACIB – Austrian Centre of Industrial Biotechnology, Petersgasse 14, 8010 Graz, Austria

[4]BioTechMed-Graz, Austria

## Supplementary Information

10 figures

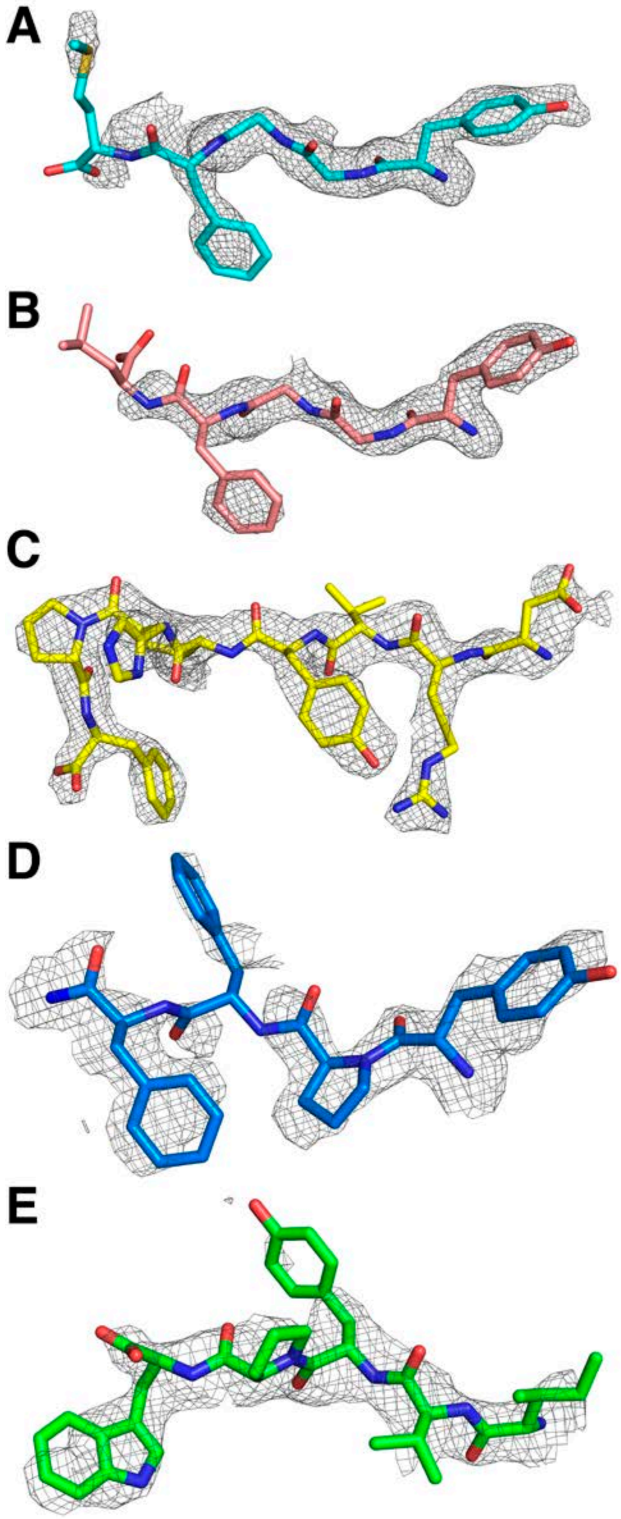


**Figure S1:** Omit difference electron density contoured at 2.5σ for the ligands. (**A**) Met-enkephalin (cyan), (**B**) Leu-enkephalin (pink), (**C**) angiotensin-II (yellow), (**D**) endomorphin-2 (blue) and (**E**) IVYPW (green). The figure was prepared using the program PyMol (http://www.pymol.org/).

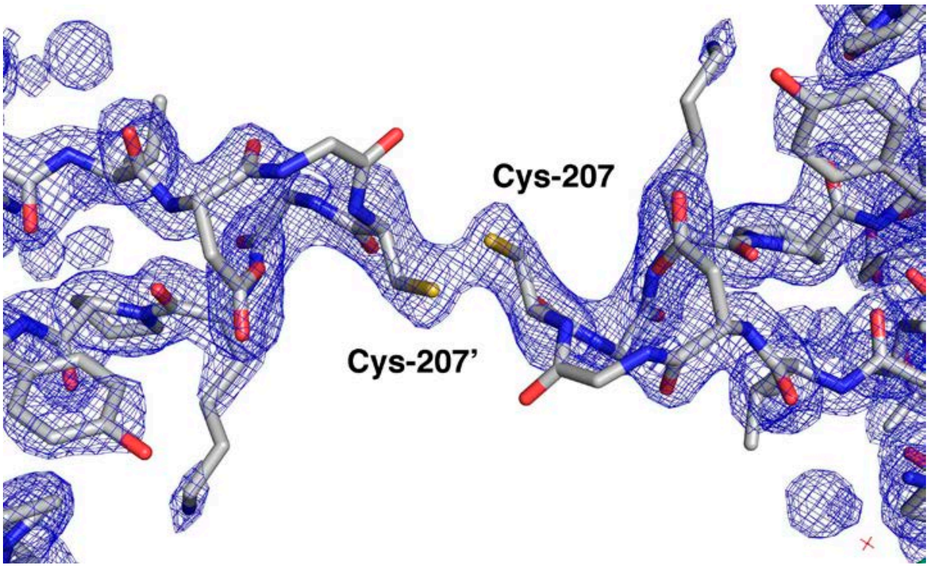


**Figure S2:** Electron density (2Fo-Fc map contoured at 1 σ) around Cys207 indicating the formation of an inter-molecular disulfide bridge with the same cysteine residue from a symmetry equivalent molecule in the crystal. The figure was prepared using the program PyMol (http://www.pymol.org/).

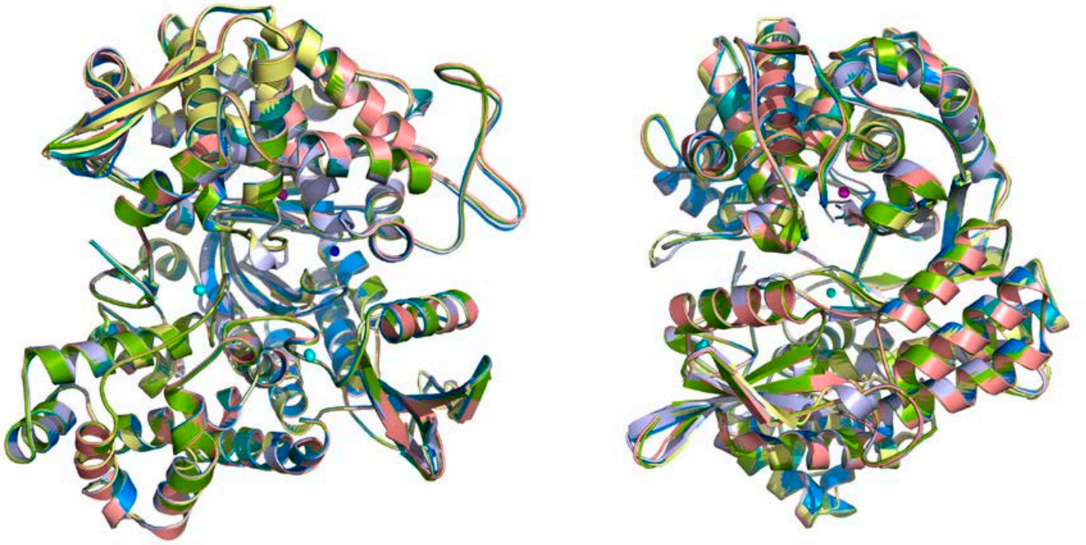

**Figure S3**: Superposition of hDPP III structures: angiotensin II (light blue), Met-enkephalin (yellow), Leu-enkephalin (deep teal), endomorphin-2 (blue), IVYPW (pink) and the unbound hDPP III in the closed conformation (green). Zinc, magnesium and potassium ions are shown as purple, cyan and blue spheres, respectively. The two views are related to each other by a 90° rotation around the vertical axis. The figure was prepared using the program PyMol (http://www.pymol.org/).

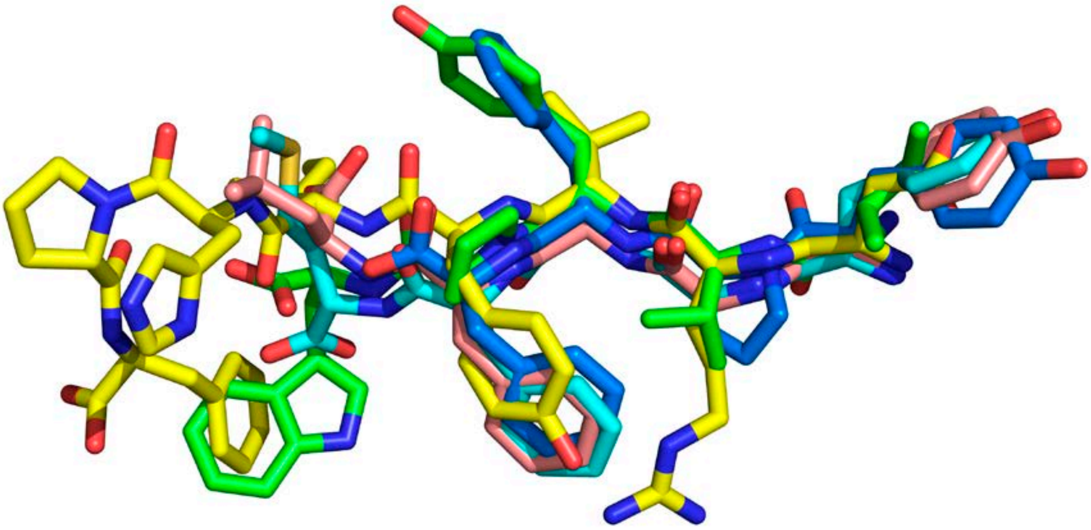

**Figure S4**: Superposition of the bound ligands (based on the superposition of the protein chains, see Figure S3): Met-enkephalin (cyan), Leu-enkephalin (pink), IVYPW (green), endomorphin-2 (blue) and angiotensin-II (yellow). The figure was prepared using the program PyMol (http://www.pymol.org/).

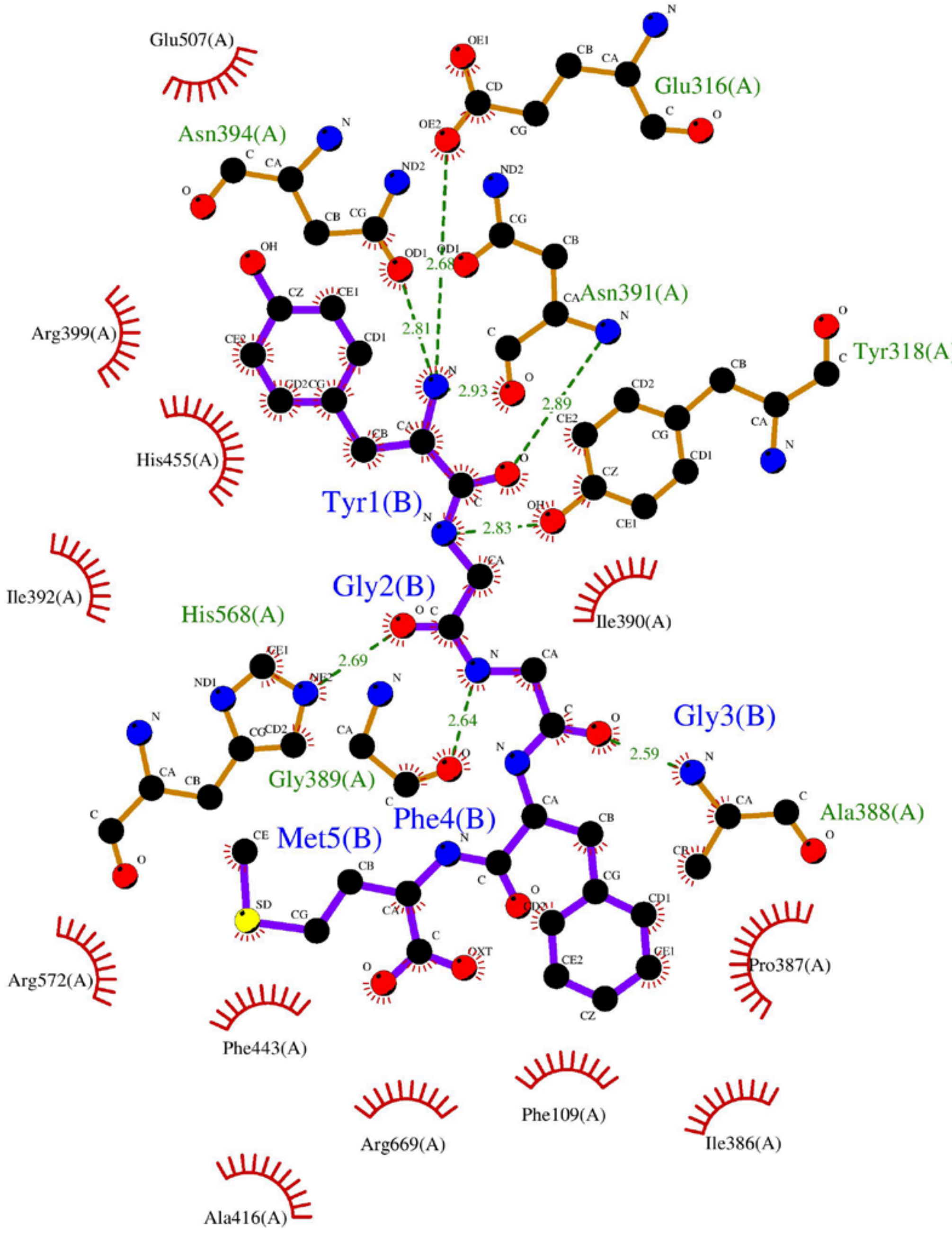


**Figure S5:** LigPlot-diagram (http://www.ebi.ac.uk/thornton-srv/software/LIGPLOT/) of the interactions of Met-enkephalin (magenta) with hDPP III.

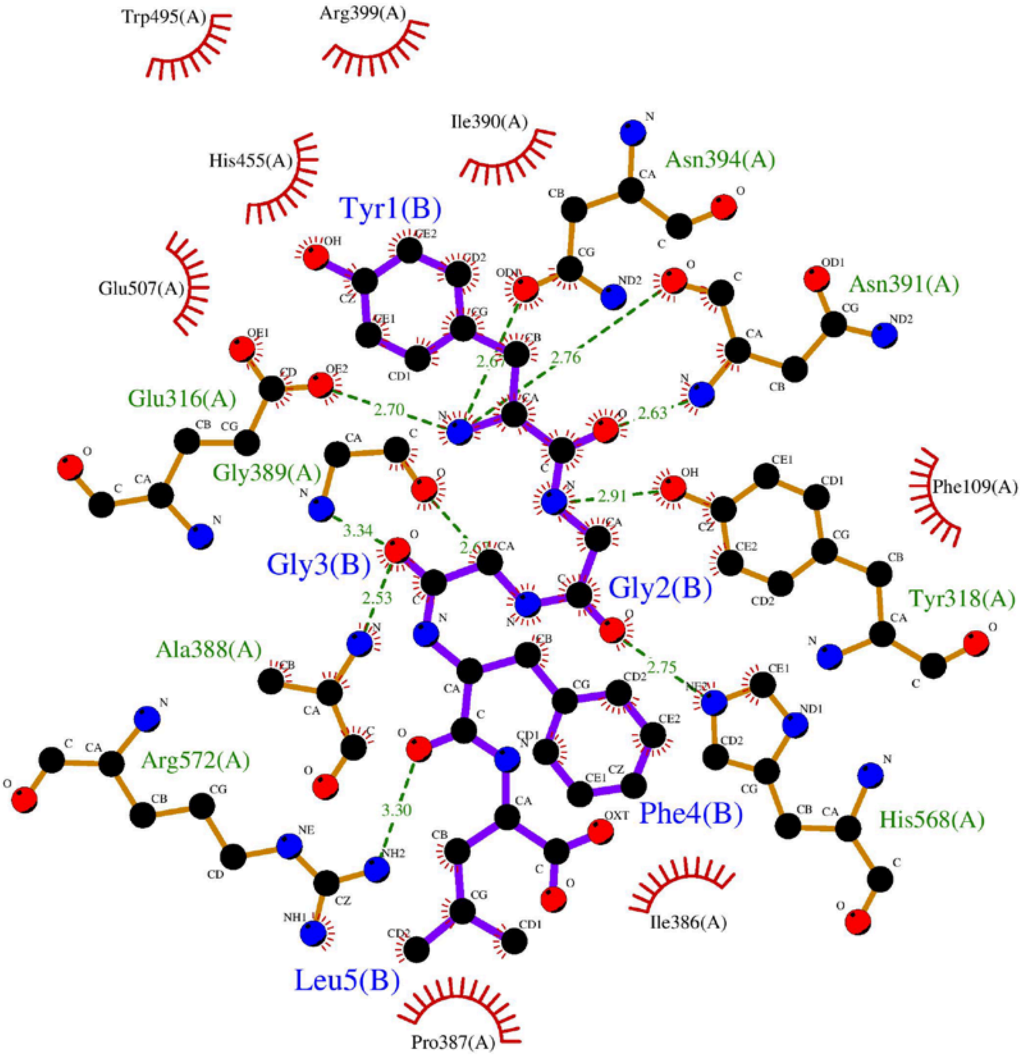


**Figure S6:** LigPlot-diagram (http://www.ebi.ac.uk/thornton-srv/software/LIGPLOT/) of the interactions of Leu-enkephalin (magenta) with hDPP III.

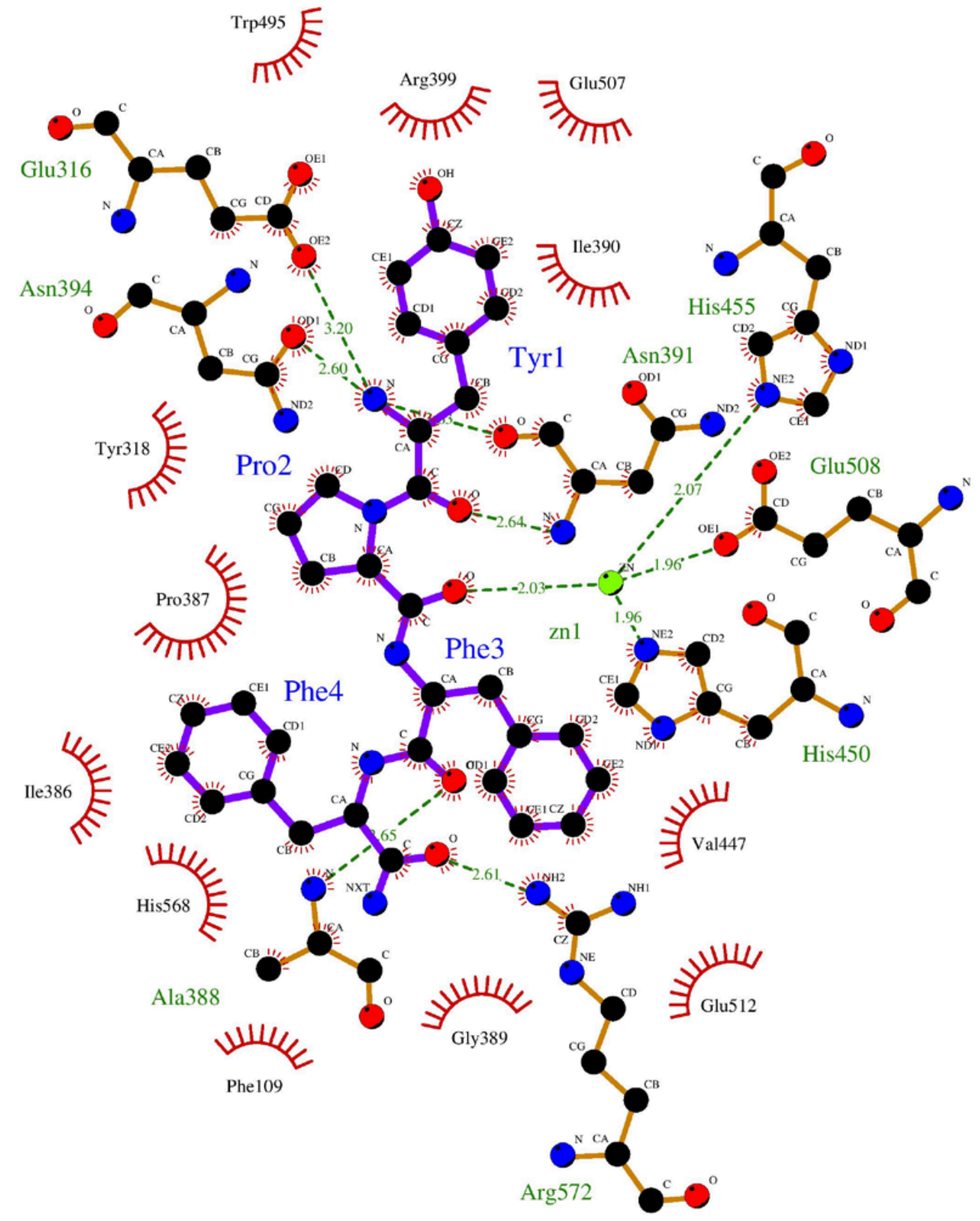


**Figure S7**: LigPlot-diagram (http://www.ebi.ac.uk/thornton-srv/software/LIGPLOT/) of the interactions of endomorphin-2 (magenta) with hDPP III.

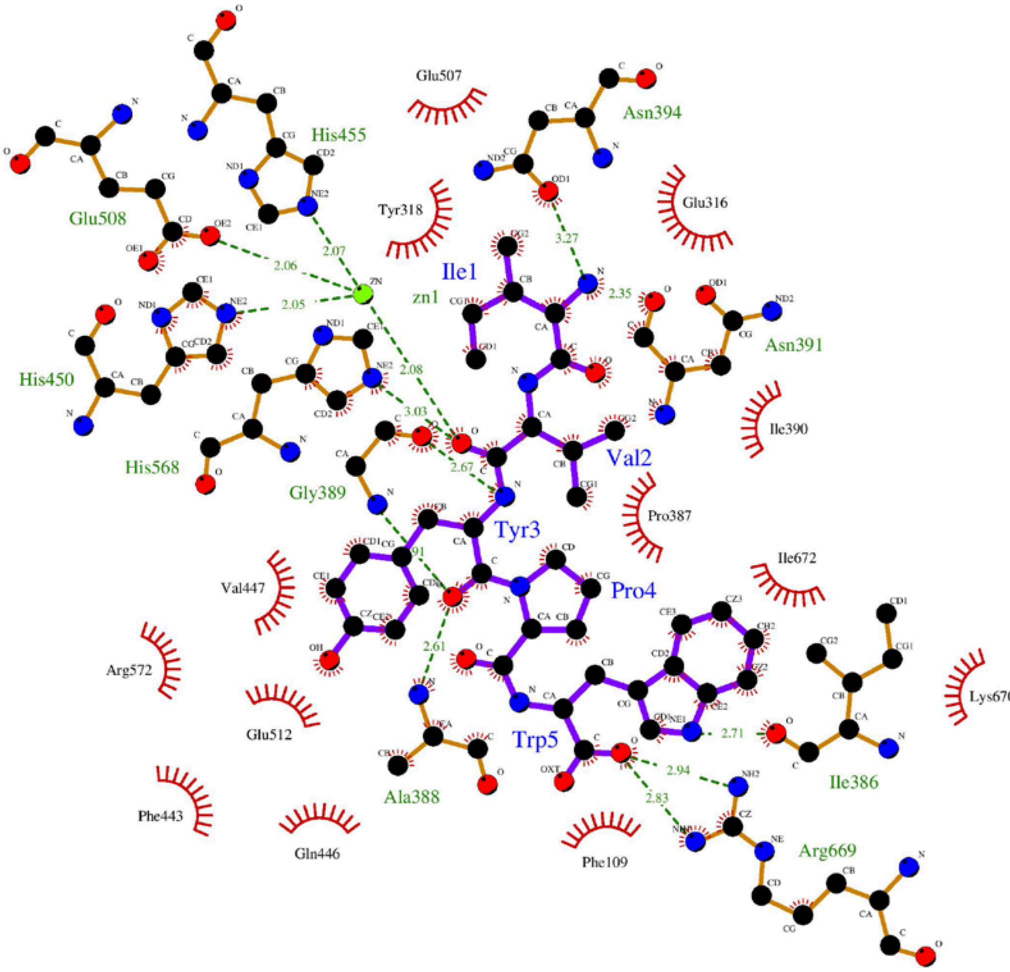


**Figure S8**: LigPlot-diagram (http://www.ebi.ac.uk/thornton-srv/software/LIGPLOT/) of the interactions of IVYPW (magenta) with hDPP III.

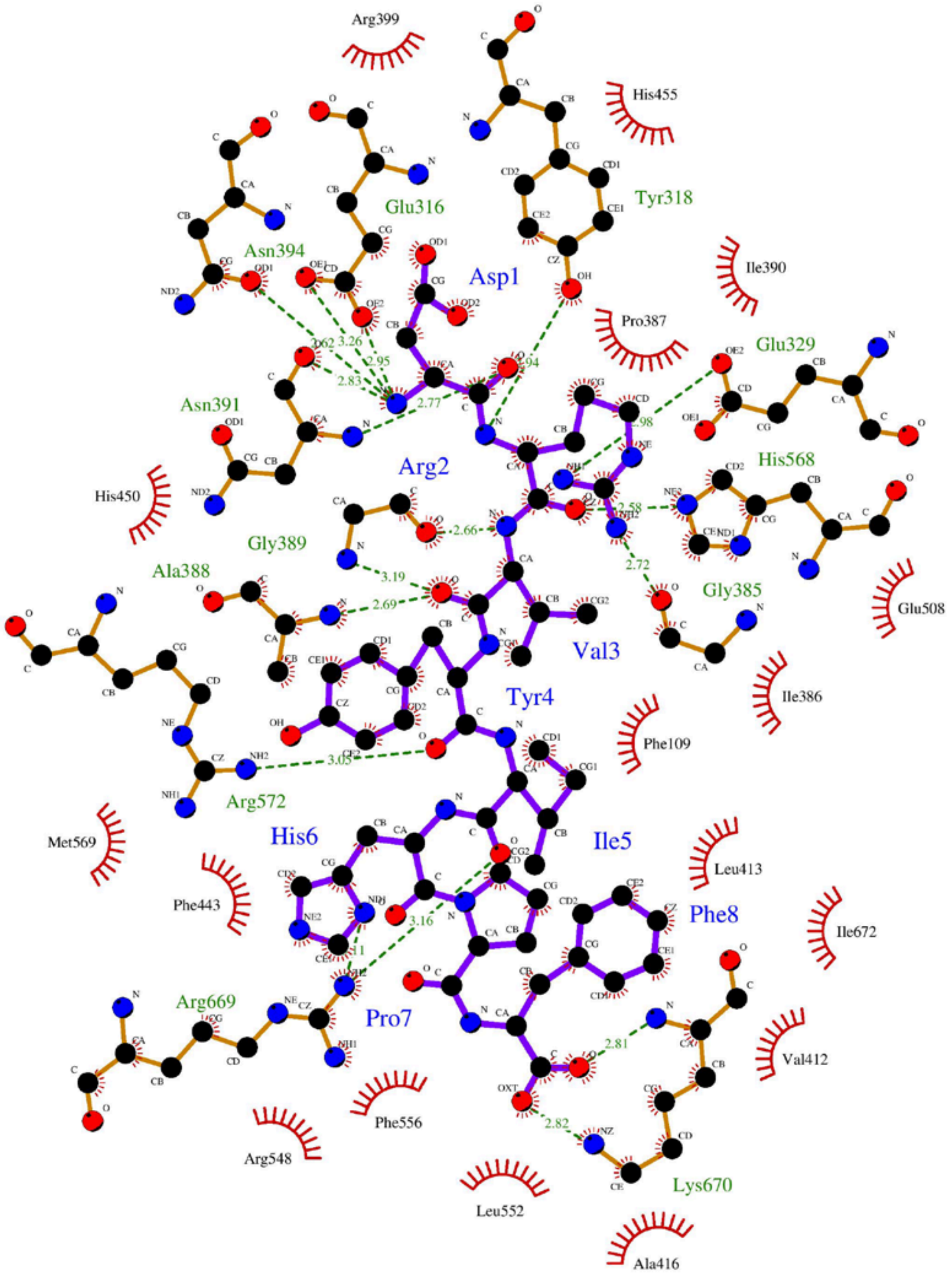


**Figure S9:** LigPlot-diagram (http://www.ebi.ac.uk/thornton-srv/software/LIGPLOT/) of the interactions of angiotensin-II (magenta) with hDPP III.

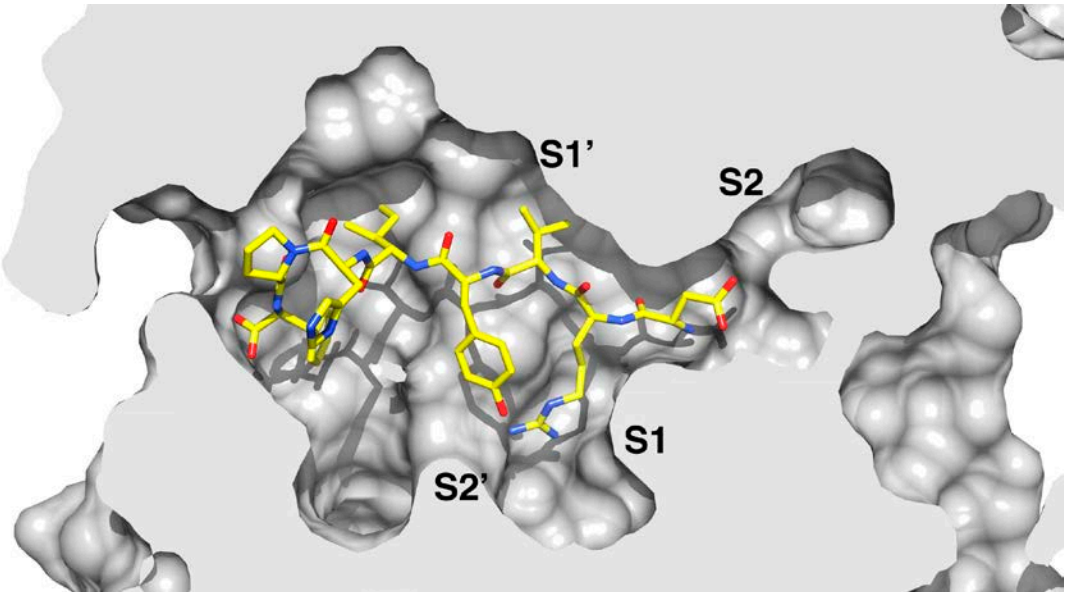


**Figure S10:** Surface representation of the binding site (vertically cut open) binding site in the hDPP III structure in complex with angiotensin-II. The peptide is shown in a sticks representation (yellow). The figure was generated using the program Chimera (http://www.cgl.ucsf.edu/chimera/).
